# Nuclear Receptors in Regulation of Mouse ES Cell Pluripotency and Differentiation

**DOI:** 10.1155/2007/61563

**Published:** 2007-08-27

**Authors:** Eimear M. Mullen, Peili Gu, Austin J. Cooney

**Affiliations:** ^1^Department of Molecular and Cellular Biology, Baylor College of Medicine, Houston, TX 77030, USA; ^2^Department of Biological Sciences, Northern Kentucky University, Highland Heights, KY 41099, USA

## Abstract

Embryonic stem (ES) cells have great therapeutic potential because they are capable of indefinite self-renewal and have the potential to differentiate into over 200 different cell types that compose the human body. The switch from the pluripotent phenotype to a differentiated cell involves many complex signaling pathways including those involving LIF/Stat3 and the transcription factors Sox2, Nanog and Oct-4. Many nuclear receptors play an important role in the maintenance of pluripotence (ERRβ, SF-1, LRH-1, DAX-1) repression of the ES cell phenotype (RAR, RXR, GCNF) and also the differentiation of ES cells (PPARγ). Here we review the roles of the nuclear receptors involved in regulating these important processes in ES cells.

## 1. INTRODUCTION

The nuclear receptor peroxisome proliferator activated receptor gamma (PPARγ) plays an important role in the differentiation of adipose cells and osteoblasts, and thus has the potential to direct embryonic stem (ES) cells to differentiate into these cell types for future therapeutic uses in disease treatment. This potential is real as nuclear receptor family members regulate many of the key functions of ES cells, and they are capable of unlimited self-renewal and can potentially differentiate into any of over 200 cell types in the body. They are derived from the inner cell mass of the mammalian blastocyst [1–4]. The pluripotency of ES cells is maintained by several key regulatory transcription factors and signaling molecules, which establish precise patterns of gene expression that are characteristics of the undifferentiated phenotype of ES cell [5]. Some of these key regulators are leukemia inhibitory factor (LIF) and the transcription factors Oct-4, Sox2 and, Nanog [5]. LIF belongs to the interleukin-6 cytokine family and binds to a heterodimeric receptor, which then leads to activation of the Jak/Stat pathway. Activation of Stat3 is essential and sufficient to maintain the mouse ES cell pluripotence, however, the LIF STAT3 pathway is mouse specific (related to diapause) and does not play a role in human embryonic stem cells [6–8]. Wnt3A is also important in the maintenance of ES cell pluripotence [9]. It was found that its presence in the media can maintain the pluripotent nature of ES cells, but it appears that this action occurs synergistically with LIF [10].

Oct-4 is a member of the POU homeodomain family of transcription factors, which acts as a gatekeeper to prevent ES cell differentiation by maintaining pluripotent gene expression and inhibiting expression of lineage determination factors. When repressed or inactivated in ES cells, differentiation occurs along the trophoectodermal lineage. Over expression of Oct-4 causes ES cells to differentiate mainly into primitive endoderm-like derivatives [11]. These divergent effects of Oct-4 suggest that it regulates the transcription of genes involved in coordination of multiple cellular functions and early cell fate decisions. Oct-4 usually binds to the octamer DNA sequence ATGCAAAT in ES cell-specific genes, and this binding often occurs in conjunction with Sox2 (a member of The SRY HMG box family), which binds to a neighboring Sox element [12, 13]. Nanog is an NK2 class homeobox transcription factor that was identified as a factor, which when over expressed, can maintain pluripotency even in the absence of LIF. Nanog-null embryos fail shortly after implantation, and at first give rise to pluripotent cells but these quickly differentiate along the extraembryonic endoderm lineage [14, 15].

It has been proposed that there are two mechanisms by which transcription factors play a role in the maintenance of pluripotency. First, Boyer et al. showed that the Oct-4, SOX2 and, Nanog co-occupy a substantial proportion of their target genes, which are mainly transcription factors. In addition, Oct-4, SOX2, and Nanog collaborate to form regulatory circuitry consisting of regulatory and feed forward loops that lead to coordinated auto regulation of their own expression [16]. Second, Ivanova et al. showed that the transcription factor ERRβ, along with TBX3 and TCL1 can also regulate pluripotency in ES cells, independently of the regulation by Oct-4, Sox2, and Nanog, thus forming a second regulatory axis [17].

ERRβ is a member of the nuclear receptor gene superfamily of ligand activated transcription factors [18–20]. The nuclear receptor gene superfamily includes a related, but diverse, array of transcription factors; which include nuclear hormone receptors such as the steroid receptors (NHRs) and orphan nuclear receptors [21]. NHRs are receptors for which hormonal ligands have been identified, whereas orphan receptors are so named because their ligands are unknown, at least at the time the receptor is identified. Nuclear receptors share structural motifs and domains that determine their function: a central DNA binding domain (DBD), an intervening hinge region, and a carboxy-terminal ligand binding domain (LBD), which mediates ligand-induced transactivation and participates in receptor dimerization. Nuclear receptors can exist as monomers, or homo- or heterodimers with each partner binding to specific sequences that exist as half sites separated by variable length nucleotide spacers between direct or inverted half-site repeats [22–24]. ERRβ is not the only nuclear receptor that has been implicated in regulation of ES cells, here we review the contributions of other nuclear receptors to the maintenance of pluripotency, repression of the ES cell phenotype during differentiation, and differentiation of ES cells.

### 1.1. Nuclear receptor contribution to the maintenance of pluripotence

#### 1.1.1. ERRβ (NR3B2)

The ERR subfamily of nuclear receptors consists of 3 members, ERRα, ERRβ, and ERRγ. They display a high degree of homology within their DBDs and LBDs, which indicates that they probably bind to similar ligands and target the same promoters and/or enhancers [25–29]. ERRα is broadly expressed in both the developing embryo and in the adult [30–32]. ERRβ is expressed in the developing placenta in a subset of cells in extraembryonic endoderm destined to become the chorion. Knockout mice of ERRβ have impaired trophoblast stem cell differentiation and the placenta fails to develop normally [33, 34]. ERRβ is highly restricted in the adult, being detected at low levels in the liver, stomach, skeletal muscle, heart, and kidney [25, 27]. Interestingly, Ivanova et al. identified ERRβ as having a role in the maintenance of pluripotency. Although an ES cell-based phenotype is not observed in the ERRβ KO, this might be due to maternal contribution of protein, as it is expressed in the ovulated egg or due to redundancy of expression with either ERRα or ERRγ, which would be lost in cultured ES cells. They assessed the loss of various proteins on ES cell capacity for self-renewal. Upon loss of ERRβ by shRNA knockdown, ES cells differentiated suggesting that ERRβ appeared necessary to repress differentiation. Similar studies with TBX3 and TCL1 showed similar results and microarray analysis of gene alterations in the absence these factors identified a significant overlapping set of genes. Expression of 272 genes was up regulated by the loss of ERRβ, TBX3, or TCL1. This set of genes was distinct from those regulated by Oct-4, Sox2, and Nanog. In the same set of experiments microarray analysis showed that expression of 474 genes was either up or down regulated by knockdown of Nanog, Oct4, or Sox2 but unaffected by knockdown of ERRβ, TBX3, or TCL1. This data provides evidence that two independent transcriptional pathways are operating in ES cells. The first is controlled by Oct-4, Sox2, and Nanog and could be mainly responsible for maintenance of pluripotency and repression of differentiation. The second pathway involving ERRβ, TBX3, and TCL1 seems to be responsible for repression of differentiation along specific cell lineages. However, there appears to be cross-talk between the two pathways since slight over expression of Nanog compensated for loss of ERRβ, TBX3, and TCL1 [17]. Wang et al. also identified ERRβ as interacting with Nanog [35]. However, Sauter et al. showed that there was no change in ERRβ levels when cells are induced to differentiate upon removal of LIF [36]. Since ERRα and ERRγ are involved in regulating metabolism and mitochondrial activities, it is possible that ERRβ might not be involved in maintenance of pluripotency directly but alternatively may play a role in regulating ES cell metabolism [25, 28–31, 33, 34].

#### 1.1.2. SF-1 (NR5A1)

Steroidogenic Factor 1 (SF-1; NR5A1), an orphan nuclear receptor, is, as its name suggests, expressed in steroidogenic tissues. SF-1 constitutively expressed in all three layers of the adrenal cortex, testis Leydig, and Sertoli cells, placenta, pituitary, and the hypothalamus [37, 38]. It has been shown to regulate the expression of each of the steroidogenic cytochrome P450 enzyme genes involved in steroid production [39–47], Mullerian inhibitory substance [48], and the alpha and beta subunits of the gonadotropins [49–53]. It is expressed in the urogenital ridge as early as day 9 of embryogenesis and displays dynamic expression profile in the developing gonads [37]. Disruption of SF-1 in mice leads to complete lack of adrenal glands and gonads due to adrenal and gonadal agenesis [38, 54]. A combination of the data shows that SF-1 has a central role in the regulation of steroidogenesis, development, and reproduction. Crawford et al. showed that stable expression of SF-1, which is not expressed in ES cells, directs the cells toward a more steroidogenic phenotype, which was demonstrated by the generation of progesterone. The directed differentiation of ES cells by SF-1 did not specifically require the AF2 domain but did require the proximal ligand binding domain [55].

Although SF-1 is expressed in the inner cell mass of mouse blastocysts, it is not expressed in mES cell lines. However, it was noted that the proximal promoter of murine Oct-4 contains a consensus SF-1 responsive motif (PyCAAGGpyCPu). SF-1 was found to bind to this sequence and activate transcription in embryonic carcinoma (EC) cell lines P19 and NCCIT cells, where it is expressed [56, 57]. SF-1 and Oct-4 are coexpressed in these cell lines and when SF-1 is over expressed there is approximately a 3-fold increase in Oct-4 promoter activity in NCCIT cells. It was found that there are 3 putative SF-1 sites in the human Oct-4 promoter and that one SF-1 binding site in the evolutionarily conserved region 1 (CR1) was primarily responsible for SF-1-mediated transcription of the human Oct-4 promoter. Differentiation of these EC cells with retinoic acid (RA) causes a loss in expression of both SF-1 and Oct-4, thus indicating the role of SF-1 in the maintenance of pluripotency in EC cells [56, 57].

#### 1.1.3. LRH-1 (NR5A2)

Comparison of SF-1 and Oct-4 knockout mouse models suggests that although SF-1 can regulate Oct-4 expression in EC cells, it is essential only in late organogenesis, therefore there must be another factor that compensates for SF-1 to maintain Oct-4 expression during early embryogenesis [56]. The orphan nuclear receptor liver receptor homolog-1 (LRH-1; NR5A2) is closely related to SF-1 particularly in its DNA binding domain and has the same DNA response element as SF-1 [58]. LRH-1 is expressed in endoderm derived tissues such as the liver, pancreas, and the intestines in the adult and in developing embryos [58–60]. It is involved in bile acid metabolism [61–63] and plays a role in liver development by activating genes such as HNF4α, HNF1α, and HNF3β, which coordinate hepatic gene expression [64, 65]. Like SF-1, LRH-1 also regulates the expression of genes involved in steroidogenesis. Importantly, LRH-1 is expressed at the inner cell mass of the blastocyst, in the embryonic ectoderm at the epiblast stage of embryonic development. Inactivation results in death at day 6.5 before the initiation of liver development [66]. In contrast to SF-1, which is expressed in EC cells, LRH-1 is expressed in ES cells. Upon differentiation with RA, Oct-4, and LRH-1 expression is down regulated. LRH-1 was found to bind to response elements in both the Oct-4 proximal promoter and proximal enhancer, which are evolutionarily conserved and activate its transcription. LRH-1 KO mice die at embryonic days 6.5–9.5 depending on the model analyzed. Gu et al. observed a penetrant phenotype with no embryos detected at day 7.5. However, Labelle-Dumais et al. observed a less-penetrant phenotype. Oct-4 is expressed in LRH-1^−/−^ ES cells. However, upon RA differentiation, Oct-4 expression is more rapidly lost than in WT ES cells. Sox2, FGF4, UTF1, and REX1, which are regulated by Oct 4 and function in conjunction with it in ES cells, are also more rapidly lost in LRH-1 KO ES cells than in WT cells. The decreased expression of these genes is unlikely to be a direct result of LRH-1 as they contain no putative LRH-1 binding sites in their promoters and is most likely indirect due to the precocious loss of Oct-4 expression [56]. Maintenance of Oct-4 expression is probably not the only function of LRH-1 in ES cells, there are likely too numerous other target genes. For example, in intestinal stem cells, LRH-1 and β-catenin synergistically play an important role in regulating proliferation through direct interaction and regulation of cyclin G1 expression [67]. Inactivation of the β-catenin gene is embryonic lethal at the same stage as LRH-1 and presents a similar phenotype. Thus, LRH-1 and β-catenin may cooperate to regulate ES cell proliferation and expansion from an ICM in the blastocyst to a pregastrulation epiblast [68]. Recently, it has also been found that a novel promoter directs expression of LRH-1 in ES cells and hence a novel transcript with the first ATG start codon being in exon 3 of the regular LRH-1 transcript. The novel and regular transcripts have partially overlapping tissue distribution but have important temporal and spatial differences [69]. Thus, the ES cell LRH-1 isoform may have different transcriptional properties from other isoforms of LRH-1.

#### 1.1.4. DAX1 (NR0B1)

DAX1, which stands for dosage sensitive sex reversal (DSS), adrenal hypoplasia congenital (AHC), locus on the X chromosome, gene 1, is another orphan nuclear receptor that appears to be critical in early embryonic development [70]. In contrast to canonical nuclear receptors, which have both a DBD and an LBD, DAX1 contains only an LBD. In the N-terminus there are 4 repeats purported to act as a DBD by binding to stem loop structures [70–72]. DAX1 has a known role in the establishment and maintenance of steroid producing tissues such as the testis and the adrenal cortex [73, 74]. DAX1 and SF-1 were shown to have a colocalized tissue expression in developing tissues [75, 76] and it has been shown that DAX1 acts as a repressor of SF-1 in these tissues. This transcriptional repression seems to involve direct protein-protein interactions between DAX1 and DNA-bound SF-1 via the DAX1 N-terminal domain and with subsequent recruitment of corepressors to the promoters of target genes via a DAX1 c-terminal transcriptional silencing domain [77, 78]. DAX1 has also been shown to repress LRH-1, ER, AR, and PR expression [79]. However, in contrast to molecular studies a genetic analysis of SF-1 and DAX1 in gonad development showed that rather than DAX1 antagonizing the function of SF-1 it worked in concert with it to maintain Cyp17 expression [80]. Generation of a DAX1 KO mouse model presented some problems as the gene is X-linked. The failure to generate a DAX1 knockout mouse suggests that DAX1 plays an earlier role in embryogenesis than just steroidogenesis. DAX1 was found to be expressed in early preimplantation embryos as well as in ES cells [81]. Differentiation of ES cells with RA caused a decrease in the expression of DAX1 similar to that observed for Oct-4. Disruption of the expression of DAX1 by RNA interference as well as a conditional knockout in ES cells caused their differentiation [82]. DAX-1 has been further implicated in the maintenance of pluripotence since it was discovered that it interacted with Nanog. Knockdown of DAX1 using shRNAs led to a loss of pluripotence in ES cells [35].

### 1.2. Nuclear receptor mediated repression of the ES cell phenotype

During ES cell differentiation two events must occur; one is a loss of the original phenotype and two is the induction of a new phenotype. Nuclear receptors play a role in both down regulation of the ES cell phenotype and the induction of a new cell fate.

#### 1.2.1. RARs and RXRs (NR1B1-3 and NR2B1-3)

The retinoid receptors play a prominent role in RA-mediated differentiation of ES cells. There are three genes encoding Retinoic Acid Receptors (RARα, β and γ), which bind both all-*trans* RA and 9-*cis* RA and in response activate target gene expression [83, 84]. There are also three genes encoding Retinoid X receptors (RXRα, β, and γ), which bind 9-*cis* retinoic acid (9-*cis* RA) and activate target gene expression. RARs form functional heterodimers with RXRs [21]. Gene targeting experiments in mice provided evidence that the RXR/RAR heterodimer transduces the retinoid signal during mouse development [85]. RXR enhances RAR's efficiency of binding to RA response elements (RAREs), the specificity of RARE recognition, and modulate RAR signaling [86, 87]. Work in the EC cell line PCC7 suggested that RXRα and RARγ are required for endodermal differentiation. Zechel found that selective agonists of RARα, β, and γ cause the down regulation of Oct-4, up regulation of GCNF, and the induction of neuronal markers although these agonists had distinct efficacy indicating a differential requirement of RAR isotypes during the initial stages of neuronal differentiation [88]. Since absence of RXR is embryonic lethal in mice due to myocardial malformation, it is possible that RXR plays a role in the differentiation of ES cells into cardiomyocytes. Honda et al. found that the number of beating cardiomyocytes was increased significantly following treatment with the agonist PA024 in the absence of serum and that the number was significantly decreased in the presence of the antagonist PA452, suggesting that RXR signaling regulates cardiomyocyte numbers during ES cell differentiation and maybe in normal development [89].

Early development is RA sensitive, yet thyroid hormone Receptor alpha (TRα) is expressed along with the RARs. Loss of TRα in mouse ES cells led to an increase in basal and RA-induced expression [90]. This combined with transient transfection experiments of RA responsive elements showed that TR inhibits RA-responsive gene expression and modulates RA-stimulated neural differentiation in ES cells [90].

Treatment of ES cells with RA induces not only differentiation but also repression of pluripotency genes such as Oct-4. Although there is evidence for direct regulation of Oct-4 expression by RARs in P19 cells, the inhibition of Oct-4 by RA is likely indirect. Treatment of P19 cells with RA induces expression of the orphan receptor COUP-TF, which can bind to a hormone response element in the Oct-4 proximal promoter that overlaps with the LRH-1 element. However, the expression and binding of COUP-TF occurs late in the differentiation process, after Oct-4 has been repressed. Thus, COUP-TFs are not likely to physiological mediator of Oct-4 repression in response to RA treatment [91, 92].

## 2. GCNF (NR6A1)

In contrast to COUP-TFs the orphan nuclear receptor germ cell nuclear factor (GCNF) is induced early during P19 cell differentiation and thus was a likely candidate for Oct-4 repression. GCNF is involved in regulating early embryonic development and reproduction [93–96]. It is essential for embryonic survival, normal development of the anterior-posterior axis as well as organogenesis [95, 97]. In the adult female, GCNF mRNA was detected in the growing oocytes but not in oocytes in primordial follicles, suggesting a role in oogenesis [94,98,99]. It also appears to play a role in spermatogenesis and its expression is restricted to certain stages of spermatogenesis [99]. GCNF-deficient mouse embryos die at 10.5 dpc due to cardiovascular defects and failure to establish the correct chorioallantoic connection [95]. One of the molecular defects in the GCNF KO embryos is an inability to repress and silence the Oct-4 gene [30]. In GCNF knockout embryos Oct-4 expression was present in both the primordial germ cells after gastrulation (normal) and in somatic cells (abnormal). There was also no repression of Nanog in these embryos [100].

GCNF expression is induced in response to RA treatment in P19 and ES cells and it binds to an evolutionarily conserved DR0 element in the Oct-4 proximal promoter [100]. Recombinant GCNF can bind to DNA as a monomer, homodimer, or heterodimer [101]. Dimerization of GCNF is DNA-dependent and is initiated upon binding to a DR0 element. However, endogenous GCNF induced by RA in ES cells and EC cells forms a slower migrating form of GCNF; that was shown not to be a homodimer but instead is composed of a GCNF hexamer [100, 101]. This hexamer is termed the transiently retinoid-induced factor (TRIF), which binds to and represses transcription from the DR0 on the Oct-4 promoter [96,100,102]. The expression pattern of GCNF inversely correlates with that of Oct-4 and Nanog in mouse embryos, P19 cells, and ES cells. Generation of GCNF^−/−^ ES cells showed that GCNF is required to repress the expression of Oct-4, Nanog, and Sox2 upon differentiation with RA [100]. This was a direct effect mediated through binding to DR0 elements in the Oct-4 and Nanog promoters; and likely an indirect effect on Sox2, which itself is an Oct-4 target gene [100]. Analysis of the repression mechanism of GCNF showed that it plays an essential role in the repression and silencing of Oct-4 through epigenetic modifications, especially DNA methylation. GCNF binding to the Oct-4 promoter triggers initiation of promoter DNA methylation. GCNF-dependent methylation of the Oct-4 promoter is mediated by recruitment of MBD (methylated CpG binding domain) factors, which previous studies have shown to be components of NURD repression complexes MBD3 and MBD2 and de novo DNA methyltransferases [103, 104]. In addition, GCNF interacts with DNA methyl transferase 3 (DNMT3) and likely recruits them to the Oct-4 promoter [103, 104]. The Oct-4 promoter is hypomethylated and recruitment of MBD3 and MBD2 is lost in GCNF^−/−^embryos. RNAi-mediated knockdown of MBD3 and MBD2 leads to reduced Oct-4 repression. Thus, GCNF appears to initiate repression and leads to the methylation [103, 104]. In MBD3 knockout ES cells, there is still repression of Oct-4 which is likely due to the reduction in the expression of activators such as LRH-1 after RA treatment [105, 106]. However, maintained low-level expression of Oct-4 and hypomethylation of the promoter were observed in the MBD3 KO ES cells treated with RA after six days (unpublished data AJC and PG), which means that precise repression and silencing of Oct-4 requires both GCNF and MBD3.

**Figure 1:**
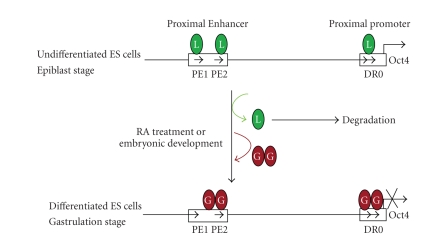
Yin-yang regulation of Oct-4 expression during ES cell differentiation by LRH-1 and GCNF, which compete for the same element. In undifferentiated ES cells LRH-1 binds to elements in the Oct-4 proximal enhancer and proximal promoter to maintain its expression during the very earliest stages of differentiation. As differentiation progresses LRH-1 expression decreases and GCNF expression is induced. At an intermediate point GCNF displaces LRH-1 and represses Oct-4 by recruiting the DNA methylation machinery that ultimately leads to the silencing of Oct-4 expression in somatic cells.

Thus, GCNF is essential for the repression of pluripotency genes such as Oct-4 and Nanog, and also in the initiation of differentiation where both transcriptional and epigenetic mechanisms play a role in its function (see Figure 1).

### 2.1. Nuclear receptor involvement in ES cell differentiation

Because of the pluripotent nature of ES cells, many nuclear receptors will, at some stage, play a role in their differentiation to anyone of the 200 cell types found in our bodies. The exact role of each nuclear receptor will depend on the cell type that the ES cells are being differentiated into. An example of the roles of nuclear receptors in ES cell differentiation is the role of the nuclear receptor PPARγ in differentiation of ES cells into adipocytes.

The peroxisome proliferator activated receptor gamma (PPARγ) is expressed in adipose, heart, kidney, spleen, intestine, colon, epithelial cells, and skeletal muscle and has been implicated in the differentiation of numerous cells and tissues including macrophages, breast, colon, and adipose [107, 108]. Targeted disruption of PPARγ is embryonic lethal and mice die and 10 dpc due to defects in the placental and cardiac development and also displays adipose tissue defects [109]. Rosen et al. showed that PPARγ is required for adipose differentiation. Analysis of PPAR$\gamma^{+/+}$ ↔ PPAR$\gamma^{-/-}$ chimeric mice revealed that the adipose tissue in these mice derived preferentially from WT cells and not the inserted PPAR$\gamma^{-/-}$ ES cells. Most other tissues had an almost even distribution of cells derived from both WT and PPAR knockout cells. They also found that when PPAR$\gamma^{-/-}$ ES cells were differentiated using a protocol to differentiate them into fat cells, no fat cells developed [110]. Vernochet et al. showed that PPARγ is expressed early in embryoid bodies and in mouse embryos at day E8.5. Addition of RA caused an increase in adipogenesis, and addition of RA and PPARγ ligand caused a further increase. However, upon addition of a PPARγ ligand alone to developing embryoid bodies overexpressing PPARγ, there was no commitment to the adipose lineage. When PPAR$\gamma^{-/-}$ embryoid bodies were differentiated, only the preadipose markers C/EBPγ and C/EBPδ were expressed. Although PPARδ was present it did not compensate for PPARγ in terminal differentiation. They proposed that PPAR is critical only in stages of adipose differentiation but is not required for early differentiation of pluripotent ES cells. The early steps of adipose differentiation are RA dependent and the latter stages are PPARγ dependent [111]. In a recent study, PPARγ expression was knocked down in ES cells using RNA interference. When the cells were induced to differentiate down an adipogenic lineage, they instead differentiated down an osteogenic lineage shown by the expression of the osteoblast markers collagen type 1, osteopontin, Cbfa1, and osteocalcin [112]. An investigation of PPARγ expression during ES cell proliferation and self-renewal showed that the PPARγ agonist 15-deoxy-$\Delta^{12,14}$-Prostaglandin J_2_(15d-PGJ2) down-regulated LIF-mediated self-renewal and proliferation and that this PPARγ-mediated regulation occurred via the JAK-STAT pathway [113].

### 2.2. Perspective

The maintenance of pluripotency and subsequent differentiations of ES cells involves a great deal of complexity. There are undoubtedly multiple mechanisms involved including signal transduction pathways and transcription factors, all of which interact to yield the phenotype of pluripotency, or of a differentiated cell. Nuclear receptors interact with these pathways and can either maintain the pluripotent phenotype, repress the acquisition of a differentiated phenotype, or aid in the acquisition of a differentiated cell type. As nuclear receptors are ligand-activated transcription factors they are part of what is now known as the druggable genome. They are obvious targets to manipulate ES cells in culture with small molecules. Based on genetic models, ligands for LRH-1 or GCNF would be predicted to affect the maintenance or repression of pluripotent gene expression mediated by these factors [56, 100] (see Figure 1). Thus, agonists for LRH-1 or antagonists for GCNF would be expected to maintain ES cell pluripotence and self-renewal, which would be optimum for large-scale culture of ES cells in the absence of LIF for therapeutic purposes. Likewise, LRH-1 antagonists or GCNF agonists would promote the silencing of pluripotency genes like Oct-4 and Nanog, which would be beneficial for differentiating ES cells into target cells. Similarly nuclear receptors can be targeted by small molecules to influence ES cell differentiation along specific pathways, for example, PPARγ agonists could promote osteoblast differentiation of ES cells. The realization of the therapeutic potential of ES cells will be greatly enhanced by the application of strategies that target nuclear receptors, or other components of the druggable genome, to push these cells into the desired cell type. Much of the pioneering works in ES cells has been performed in the mouse and each significant finding and potential target needs to be validated in human ES cells.

## Figures and Tables

**Table 1:** Summary of involvement of nuclear receptors in mouse ES cell pluripotency and differentiation.

Nuclear receptor	Function
ERRβ	Maintenance of pluripotency and repression of differentiation.
	Repression of differentiation along specific cell lineages

SF-1	Maintenance of Oct-4 expression in embryonic carcinoma cells

LRH-1	Maintenance of Oct-4 expression in ES cells.
	Interaction with β-catenin may play role in cell proliferation

DAX-1	May act as a repressor of SF-1, LRH-1, ER, AR, and PR.
	Conditional KO causes loss of pluripotency and differentiation

RAR	Down regulation of Oct-4.
	Upregulation of GCNF. Neuronal differentiation

RXR	May play role in differentiation of cardiomyocytes

GCNF	GCNF required for repression of Oct-4, Nanog, and Sox2 upon differentiation with RA.
	Repression of ES cell phenotype

PPARγ	Required in the early stages of adipose differentiation.
	Differentiation down osteogenic lineage in siRNA experiments.
	PPARγ agonist down regulated LIF-mediated self-renewal

## References

[1] Evans M, Hunter S (2002). Source and nature of embryonic stem cells. *Comptes Rendus Biologies*.

[2] Evans MJ, Kaufman MH (1981). Establishment in culture of pluripotential cells from mouse embryos. *Nature*.

[3] Gardner RL, Brook FA (1997). Reflections on the biology of embryonic stem (ES) cells. *International Journal of Developmental Biology*.

[4] Kato Y, Tsunoda Y (1993). Totipotency and pluripotency of embryonic nuclei in the mouse. *Molecular Reproduction and Development*.

[5] Boiani M, Schöler HR (2005). Regulatory networks in embryo-derived pluripotent stem cells. *Nature Reviews Molecular Cell Biology*.

[6] Burdon T, Smith A, Savatier P (2002). Signalling, cell cycle and pluripotency in embryonic stem cells. *Trends in Cell Biology*.

[7] Matsuda T, Nakamura T, Nakao K (1999). STAT3 activation is sufficient to maintain an undifferentiated state of mouse embryonic stem cells. *The EMBO Journal*.

[8] Niwa H, Burdon T, Chambers I, Smith A (1998). Self-renewal of pluripotent embryonic stem cells is mediated via activation of STAT3. *Genes and Development*.

[9] Singla DK, Schneider DJ, LeWinter MM, Sobel BE (2006). wnt3a but not wnt11 supports self-renewal of embryonic stem cells. *Biochemical and Biophysical Research Communications*.

[10] Ogawa K, Nishinakamura R, Iwamatsu Y, Shimosato D, Niwa H (2006). Synergistic action of Wnt and LIF in maintaining pluripotency of mouse ES cells. *Biochemical and Biophysical Research Communications*.

[11] Niwa H, Miyazaki J-I, Smith AG (2000). Quantitative expression of Oct-3/4 defines differentiation, dedifferentiation or self-renewal of ES cells. *Nature Genetics*.

[12] Chambers I, Smith A (2004). Self-renewal of teratocarcinoma and embryonic stem cells. *Oncogene*.

[13] Pesce M, Schöler HR (2001). Oct-4: gatekeeper in the beginnings of mammalian development. *Stem Cells*.

[14] Chambers I, Colby D, Robertson M (2003). Functional expression cloning of Nanog, a pluripotency sustaining factor in embryonic stem cells. *Cell*.

[15] Mitsui K, Tokuzawa Y, Itoh H (2003). The homeoprotein Nanog is required for maintenance of pluripotency in mouse epiblast and ES cells. *Cell*.

[16] Boyer LA, Lee TI, Cole MF (2005). Core transcriptional regulatory circuitry in human embryonic stem cells. *Cell*.

[17] Ivanova N, Dobrin R, Lu R (2006). Dissecting self-renewal in stem cells with RNA interference. *Nature*.

[18] Bardet P-L, Laudet V, Vanacker JM (2006). Studying non-mammalian models? Not a fool's ERRand!. *Trends in Endocrinology and Metabolism*.

[19] Giguère V (2002). To ERR in the estrogen pathway. *Trends in Endocrinology and Metabolism*.

[20] Horard B, Vanacker JM (2003). Estrogen receptor-related receptors: orphan receptors desperately seeking a ligand. *Journal of Molecular Endocrinology*.

[21] Olefsky JM (2001). Nuclear receptor minireview series. *Journal of Biological Chemistry*.

[22] Auwerx J, Baulieu E, Beato M (1999). A unified nomenclature system for the nuclear receptor superfamily. *Cell*.

[23] Chawla A, Repa JJ, Evans RM, Mangelsdorf DJ (2001). Nuclear receptors and lipid physiology: opening the X-files. *Science*.

[24] Lu TT, Repa JJ, Mangelsdorf DJ (2001). Orphan nuclear receptors as eLiXiRs and FiXeRs of sterol metabolism. *Journal of Biological Chemistry*.

[25] Chen F, Zhang Q, McDonald T (1999). Identification of two hERR2-related novel nuclear receptors utilizing bioinformatics and inverse PCR. *Gene*.

[26] Eudy JD, Yao S, Weston MD (1998). Isolation of a gene encoding a novel member of the nuclear receptor superfamily from the critical region of Usher syndrome type IIa at 1q41. *Genomics*.

[27] Giguère V, Yang N, Segui P, Evans RM (1988). Identification of a new class of steroid hormone receptors. *Nature*.

[28] Heard DJ, Norby PL, Holloway J, Vissing H (2000). Human ERRγ a third member of the estrogen receptor-related receptor (ERR) subfamily of orphan nuclear receptors: tissue-specific isoforms are expressed during development and in the adult. *Molecular Endocrinology*.

[29] Hong H, Yang L, Stallcup MR (1999). Hormone-independent transcriptional activation and coactivator binding by novel orphan nuclear receptor ERR3. *Journal of Biological Chemistry*.

[30] Bonnelye E, Vanacker JM, Dittmar T (1997). The ERR-1 orphan receptor is a transcriptional activator expressed during bone development. *Molecular Endocrinology*.

[31] Sladek R, Bader J-A, Giguère V (1997). The orphan nuclear receptor estrogen-related receptor or α is a transcriptional regulator of the human medium-chain Acyl coenzyme A dehydrogenase gene. *Molecular and Cellular Biology*.

[32] Vanacker JM, Bonnelye E, Delmarre C, Laudet V (1998). Activation of the thyroid hormone receptor α gene promoter by the orphan nuclear receptor ERRα. *Oncogene*.

[33] Luo J, Sladek R, Bader J-A, Matthyssen A, Rossant J, Giguère V (1997). Placental abnormalities in mouse embryos lacking the orphan nuclear receptor ERR-β. *Nature*.

[34] Pettersson K, Svensson K, Mattsson R, Carlsson B, Ohlsson R, Berkenstam A (1996). Expression of a novel member of estrogen response element-binding nuclear receptors is restricted to the early stages of chorion formation during mouse embryogenesis. *Mechanisms of Development*.

[35] Wang J, Rao S, Chu J (2006). A protein interaction network for pluripotency of embryonic stem cells. *Nature*.

[36] Sauter CN, McDermid RL, Weinberg AL (2005). Differentiation of murine embryonic stem cells induces progesterone receptor gene expression. *Experimental Cell Research*.

[37] Ikeda Y, Shen W-H, Ingraham HA, Parker KL (1994). Developmental expression of mouse steroidogenic factor-1, an essential regulator of the steroid hydroxylases. *Molecular Endocrinology*.

[38] Sadovsky Y, Crawford PA, Woodson KG (1995). Mice deficient in the orphan receptor steroidogenic factor 1 lack adrenal glands and gonads but express P450 side-chain-cleavage enzyme in the placenta and have normal embryonic serum levels of corticosteroids. *Proceedings of the National Academy of Sciences of the United States of America*.

[39] Bakke M, Lund J (1995). Mutually exclusive interactions of two nuclear orphan receptors determine activity of a cyclic adenosine $3^{\prime },5^{\prime }$-monophosphate-responsive sequence in the bovine CYP17 gene. *Molecular Endocrinology*.

[40] Clemens JW, Lala DS, Parker KL, Richards JS (1994). Steroidogenic factor-1 binding and transcriptional activity of the cholesterol side-chain cleavage promoter in rat granulosa cells. *Endocrinology*.

[41] Honda S-I, Morohashi K-I, Nomura M, Takeya H, Kitajima M, Omura T (1993). Ad4BP regulating steroidogenic P-450 gene is a member of steroid hormone receptor superfamily. *Journal of Biological Chemistry*.

[42] Ikeda Y, Lala DS, Luo X, Kim E, Moisan M-P, Parker KL (1993). Characterization of the mouse FTZ-F1 gene, which encodes a key regulator of steroid hydroxylase gene expression. *Molecular Endocrinology*.

[43] Lala DS, Rice DA, Parker KL (1992). Steroidogenic factor I, a key regulator of steroidogenic enzyme expression, is the mouse homolog of fushi tarazu-factor I. *Molecular Endocrinology*.

[44] Lynch JP, Lala DS, Peluso JJ, Luo W, Parker KL, White BA (1993). Steroidogenic factor 1, an orphan nuclear receptor, regulates the expression of the rat aromatase gene in gonadal tissues. *Molecular Endocrinology*.

[45] Michael MD, Kilgore MW, Morohashi K-I, Simpson ER (1995). Ad4BP/SF-1 regulates cyclic AMP-induced transcription from the proximal promoter (PII) of the human aromatase P450 (CYP19) gene in the ovary. *Journal of Biological Chemistry*.

[46] Morohashi K-I, Zanger UM, Honda S-I, Hara M, Waterman MR, Omura T (1993). Activation of CYP11A and CYP11B gene promoters by the steroidogenic cell-specific transcription factor, Ad4BP. *Molecular Endocrinology*.

[47] Zhang P, Mellon SH (1996). The orphan nuclear receptor steroidogenic factor-1 regulates the cyclic adenosine $3^{\prime },5^{\prime }$-monophosphate-mediated transcriptional activation of rat cytochrome P450c17 (1α7-hydroxylase/c17-20 lyase). *Molecular Endocrinology*.

[48] Shen W-H, Moore CCD, Ikeda Y, Parker KL, Ingraham HA (1994). Nuclear receptor steroidogenic factor 1 regulates the müllerian inhibiting substance gene: a link to the sex determination cascade. *Cell*.

[49] Barnhart KM, Mellon PL (1994). The orphan nuclear receptor, steroidogenic factor-1, regulates the glycoprotein hormone α-subunit gene in pituitary gonadotropes. *Molecular Endocrinology*.

[50] Halvorson LM, Kaiser UB, Chin WW (1996). Stimulation of luteinizing hormone β gene promoter activity by the orphan nuclear receptor, steroidogenic factor-1. *Journal of Biological Chemistry*.

[51] Ingraham HA, Lala DS, Ikeda Y (1994). The nuclear receptor steroidogenic factor 1 acts at multiple levels of the reproductive axis. *Genes and Development*.

[52] Keri RA, Nilson JH (1996). A steroidogenic factor-1 binding site is required for activity of the luteinizing hormone β subunit promoter in gonadotropes of transgenic mice. *Journal of Biological Chemistry*.

[53] Lee SL, Sadovsky Y, Swirnoff AH (1996). Luteinizing hormone deficiency and female infertility in mice lacking the transcription factor NGFI-A (Egr-1). *Science*.

[54] Luo X, Ikeda Y, Parker KL (1994). A cell-specific nuclear receptor is essential for adrenal and gonadal development and sexual differentiation. *Cell*.

[55] Crawford PA, Sadovsky Y, Milbrandt J (1997). Nuclear receptor steroidogenic factor 1 directs embryonic stem cells toward the steroidogenic lineage. *Molecular and Cellular Biology*.

[56] Gu P, Goodwin B, Chung AC-K (2005). Orphan nuclear receptor LRH-1 is required to maintain Oct4 expression at the epiblast stage of embryonic development. *Molecular and Cellular Biology*.

[57] Yang H-M, Do H-J, Kim D-K (2007). Transcriptional regulation of human Oct4 by steroidogenic factor-1. *Journal of Cellular Biochemistry*.

[58] Nitta M, Ku S, Brown C, Okamoto AY, Shan B (1999). CPF: an orphan nuclear receptor that regulates liver-specific expression of the human cholesterol α7-hydroxylase gene. *Proceedings of the National Academy of Sciences of the United States of America*.

[59] Annicotte J-S, Fayard E, Swift GH (2003). Pancreatic-duodenal homeobox 1 regulates expression of liver receptor homolog 1 during pancreas development. *Molecular and Cellular Biology*.

[60] Rausa FM, Galarneau L, Bélanger L, Costa RH (1999). The nuclear receptor fetoprotein transcription factor is coexpressed with its target gene HNF-3β in the developing murine liver intestine and pancreas. *Mechanisms of Development*.

[61] del Castillo-Olivares A, Campos JA, Pandak WM, Gil G (2004). The role of α1-fetoprotein transcription factor/LRH-1 in bile acid biosynthesis: a known nuclear receptor activator that can act as a suppressor of bile acid biosynthesis. *Journal of Biological Chemistry*.

[62] Fayard E, Auwerx J, Schoonjans K (2004). LRH-1: an orphan nuclear receptor involved in development, metabolism and steroidogenesis. *Trends in Cell Biology*.

[63] Schoonjans K, Annicotte J-S, Huby T (2002). Liver receptor homolog 1 controls the expression of the scavenger receptor class B type I. *EMBO Reports*.

[64] Galarneau L, Paré J-F, Allard D (1996). The α1-fetoprotein locus is activated by a nuclear receptor of the Drosophila FTZ-F1 family. *Molecular and Cellular Biology*.

[65] Paré J-F, Roy S, Galarneau L, Bélanger L (2001). The mouse fetoprotein transcription factor (FTF) gene promoter is regulated by three GATA elements with tandem E box and Nkx motifs, and FTF in turn activates the Hnf3β, Hnf4α, and Hnf1α gene promoters. *Journal of Biological Chemistry*.

[66] Paré J-F, Malenfant D, Courtemanche C (2004). The fetoprotein transcription factor (FTF) gene is essential to embryogenesis and cholesterol homeostasis and is regulated by a DR4 element. *Journal of Biological Chemistry*.

[67] Botrugno OA, Fayard E, Annicotte J-S (2004). Synergy between LRH-1 and β-catenin Induces G1 cyclin-mediated cell proliferation. *Molecular Cell*.

[68] Huelsken J, Vogel R, Brinkmann V, Erdmann B, Birchmeier C, Birchmeier W (2000). Requirement for β-catenin in anterior-posterior axis formation in mice. *Journal of Cell Biology*.

[69] Gao D-M, Wang L-F, Liu J, Kong Y-Y, Wang Y, Xie Y-H (2006). Expression of mouse liver receptor homologue 1 in embryonic stem cells is directed by a novel promoter. *FEBS Letters*.

[70] Burris TP, Guo W, McCabe ERB (1996). The gene responsible for adrenal hypoplasia congenita, DAX-1, encodes a nuclear hormone receptor that defines a new class within the superfamily. *Recent Progress in Hormone Research*.

[71] Seol W, Choi H-S, Moore DD (1996). An orphan nuclear hormone receptor that lacks a DNA binding domain and heterodimerizes with other receptors. *Science*.

[72] Zhang Z, Burch PE, Cooney AJ (2004). Genomic analysis of the nuclear receptor family: new insights into structure, regulation, and evolution from the rat genome. *Genome Research*.

[73] Guo W, Mason JS, Stone CG (1995). Diagnosis of X-linked adrenal hypoplasia congenita by mutation analysis of the DAX1 gene. *Journal of the American Medical Association*.

[74] Muscatelli F, Strom TM, Walker AP (1994). Mutations in the DAX-1 gene give rise to both X-linked adrenal hypoplasia congentia and hypogonadotropic hypogonadism. *Nature*.

[75] Ikeda Y, Swain A, Weber TJ (1996). Steroidogenic factor 1 and Dax-1 colocalize in multiple cell lineages: potential links in endocrine development. *Molecular Endocrinology*.

[76] Ikeda Y, Takeda Y, Shikayama T, Mukai T, Hisano S, Morohashi K-I (2001). Comparative localization of Dax-1 and Ad4BP/SF-1 during development of the hypothalamic-pituitary-gonadal axis suggests their closely related and distinct functions. *Developmental Dynamics*.

[77] Ito M, Yu R, Jameson JL (1997). DAX-1 inhibits SF-1-mediated transactivation via a carboxy-terminal domain that is deleted in adrenal hypoplasia congenita. *Molecular and Cellular Biology*.

[78] Lalli E, Bardoni B, Zazopoulos E (1997). A transcriptional silencing domain in DAX-1 whose mutation causes adrenal hypoplasia congenita. *Molecular Endocrinology*.

[79] Iyer AK, McCabe ERB (2004). Molecular mechanisms of DAX1 action. *Molecular Genetics and Metabolism*.

[80] Park SY, Meeks JJ, Raverot G (2005). Nuclear receptors Sf1 and Dax1 function cooperatively to mediate somatic cell differentiation during testis development. *Development*.

[81] Clipsham R, Niakan K, McCabe ERB (2004). Nr0b1 and its network partners are expressed early in murine embryos prior to steroidogenic axis organogenesis. *Gene Expression Patterns*.

[82] Niakan KK, Davis EC, Clipsham RC (2006). Novel role for the orphan nuclear receptor Dax1 in embryogenesis, different from steroidogenesis. *Molecular Genetics and Metabolism*.

[83] Allenby G, Bocquel MT, Saunders M (1993). Retinoic acid receptors and retinoid X receptors: interactions with endogenous retinoic acids. *Proceedings of the National Academy of Sciences of the United States of America*.

[84] Egea PF, Klaholz BP, Moras D (2000). Ligand-protein interactions in nuclear receptors of hormones. *FEBS Letters*.

[85] Kastner P, Mark M, Ghyselinck N (1997). Genetic evidence that the retinoid signal is transduced by heterodimeric RXR/RAR functional units during mouse development. *Development*.

[86] Chambon P (1996). A decade of molecular biology of retinoic acid receptors. *The FASEB Journal*.

[87] Giguere V (1994). Retinoic acid receptors and cellular retinoid binding proteins: complex interplay in retinoid signaling. *Endocrine Reviews*.

[88] Zechel C (2005). Requirement of retinoic acid receptor isotypes α, β, and γ during the initial steps of neural differentiation of PCC7 cells. *Molecular Endocrinology*.

[89] Honda M, Hamazaki TS, Komazaki S, Kagechika H, Shudo K, Asashima M (2005). RXR agonist enhances the differentiation of cardiomyocytes derived from embryonic stem cells in serum-free conditions. *Biochemical and Biophysical Research Communications*.

[90] Lee L-R, Mortensen RM, Larson CA, Brent GA (1994). Thyroid hormone receptor-α inhibits retinoic acid-responsive gene expression and modulates retinoic acid-stimulated neural differentiation in mouse embryonic stem cells. *Molecular Endocrinology*.

[91] Ben-Shushan E, Sharir H, Pikarsky E, Bergman Y (1995). A dynamic balance between ARP-1/COUP-TFII, EAR-3/COUP-TFI, and retinoic acid receptor:retinoid X receptor heterodimers regulates Oct-3/4 expression in embryonal carcinoma cells. *Molecular and Cellular Biology*.

[92] Pikarsky E, Sharir H, Ben-Shushan E, Bergman Y (1994). Retinoic acid represses Oct-3/4 gene expression through several retinoic acid-responsive elements located in the promoter-enhancer region. *Molecular and Cellular Biology*.

[93] Lan Z-J, Gu P, Xu X, Cooney AJ (2003). Expression of the orphan nuclear receptor, germ cell nuclear factor, in mouse gonads and preimplantation embryos. *Biology of Reproduction*.

[94] Lan Z-J, Gu P, Xu X (2003). GCNF-dependent repression of BMP-15 and GDF-9 mediates gamete regulation of female fertility. *The EMBO Journal*.

[95] Chung AC-K, Katz D, Pereira FA (2001). Loss of orphan receptor germ cell nuclear factor function results in ectopic development of the tail bud and a novel posterior truncation. *Molecular and Cellular Biology*.

[96] Fuhrmann G, Chung AC-K, Jackson KJ (2001). Mouse germline restriction of Oct4 expression by germ cell nuclear factor. *Developmental Cell*.

[97] David R, Joos TO, Dreyer C (1998). Anteroposterior patterning and organogenesis of Xenopus laevis require a correct dose of germ cell nuclear factor (xGCNF). *Mechanisms of Development*.

[98] Mehta DV, Kim Y-S, Dixon D, Jetten AM (2002). Characterization of the expression of the retinoid-related, testis-associated receptor (RTR) in trophoblasts. *Placenta*.

[99] Zechel C (2005). The germ cell nuclear factor (GCNF). *Molecular Reproduction and Development*.

[100] Gu P, LeMenuet D, Chung AC-K, Mancini M, Wheeler DA, Cooney AJ (2005). Orphan nuclear receptor GCNF is required for the repression of pluripotency genes during retinoic acid-induced embryonic stem cell differentiation. *Molecular and Cellular Biology*.

[101] Gu P, Morgan DH, Sattar M (2005). Evolutionary trace-based peptides identify a novel asymmetric interaction that mediates oligomerization in nuclear receptors. *Journal of Biological Chemistry*.

[102] Schmitz TP, Süsens U, Borgmeyer U (1999). DNA binding, protein interaction and differential expression of the human germ cell nuclear factor. *Biochimica et Biophysica Acta*.

[103] Gu P, Le Menuet D, Chung AC-K, Cooney AJ (2006). Differential recruitment of methylated CpG binding domains by the orphan receptor GCNF initiates the repression and silencing of Oct4 expression. *Molecular and Cellular Biology*.

[104] Sato N, Kondo M, Arai K-I (2006). The orphan nuclear receptor GCNF recruits DNA methyltransferase for Oct-3/4 silencing. *Biochemical and Biophysical Research Communications*.

[105] Kaji K, Caballero IM, MacLeod R, Nichols J, Wilson VA, Hendrich B (2006). The NuRD component Mbd3 is required for pluripotency of embryonic stem cells. *Nature Cell Biology*.

[106] Kaji K, Nichols J, Hendrich B (2007). Mbd3, a component of the NuRD co-repressor complex, is reqiured for development of pluripotent cells. *Development*.

[107] Elbrecht A, Chen Y, Cullinan CA (1996). Molecular cloning, expression and characterization of human peroxisome proliferator activated receptors γ1 and γ2. *Biochemical and Biophysical Research Communications*.

[108] Mukherjee R, Jow L, Croston GE, Paterniti JR (1997). Identification, characterization, and tissue distribution of human peroxisome proliferator-activated receptor (PPAR) isoforms PPARγ2 versus PPARγ1 and activation with retinoid X receptor agonists and antagonists. *Journal of Biological Chemistry*.

[109] Barak Y, Nelson MC, Ong ES (1999). PPARγ is required for placental, cardiac, and adipose tissue development. *Molecular Cell*.

[110] Rosen ED, Sarraf P, Troy AE (1999). PPARγ is required for the differentiation of adipose tissue in vivo and in vitro. *Molecular Cell*.

[111] Vernochet C, Milstone DS, Iehlé C (2002). PPARγ-dependent and PPARγ-independent effects on the development of adipose cells from embryonic stem cells. *FEBS Letters*.

[112] Yamashita A, Takada T, Nemoto K-I, Yamamoto G, Torii R (2006). Transient suppression of PPARγ directed ES cells into an osteoblastic lineage. *FEBS Letters*.

[113] Rajasingh J, Bright JJ (2006). 15-deoxy-$\Delta^{12,14}$-prostaglandin ${\text{J}}_{2}$ regulates leukemia inhibitory factor signaling through JAK-STAT pathway in mouse embryonic stem cells. *Experimental Cell Research*.

